# Clinical and Preclinical Outcomes of Combining Targeted Therapy With Radiotherapy

**DOI:** 10.3389/fonc.2021.749496

**Published:** 2021-10-18

**Authors:** May Elbanna, Nayela N. Chowdhury, Ryan Rhome, Melissa L. Fishel

**Affiliations:** ^1^ Department of Radiation Oncology, Indiana University School of Medicine, Indianapolis, IN, United States; ^2^ Indiana University Simon Comprehensive Cancer Center, Indiana University School of Medicine, Indianapolis, IN, United States; ^3^ Department of Pharmacology and Toxicology, Indiana University School of Medicine, Indianapolis, IN, United States; ^4^ Department of Pediatrics and Herman B Wells Center for Pediatric Research, Indiana University School of Medicine, Indianapolis, IN, United States

**Keywords:** cancer, DNA damage, combination (combined) therapy, radiation therapy, radiosenisitizing agent, targeted therapy

## Abstract

In the era of precision medicine, radiation medicine is currently focused on the precise delivery of highly conformal radiation treatments. However, the tremendous developments in targeted therapy are yet to fulfill their full promise and arguably have the potential to dramatically enhance the radiation therapeutic ratio. The increased ability to molecularly profile tumors both at diagnosis and at relapse and the co-incident progress in the field of radiogenomics could potentially pave the way for a more personalized approach to radiation treatment in contrast to the current ‘‘one size fits all’’ paradigm. Few clinical trials to date have shown an improved clinical outcome when combining targeted agents with radiation therapy, however, most have failed to show benefit, which is arguably due to limited preclinical data. Several key molecular pathways could theoretically enhance therapeutic effect of radiation when rationally targeted either by directly enhancing tumor cell kill or indirectly through the abscopal effect of radiation when combined with novel immunotherapies. The timing of combining molecular targeted therapy with radiation is also important to determine and could greatly affect the outcome depending on which pathway is being inhibited.

## Introduction

A plethora of factors are involved in the development and progression of cancer in individuals such as family history, age, sex, primary site of origin and driver mutations; thus, treatment depends upon the goal of therapy - curative or palliative. Treatment for cancer involves multiple approaches including surgery, chemotherapy, immunotherapy, small molecules that target certain cancer signaling pathways, and radiation depending on cancer type or status. The use of multiple treatments concurrently is referred to as multi-modality treatment. Radiation therapy plays a crucial role in the management of cancer. Also known as radiotherapy (RT), it is a method of impeding cancer cell division by using high-energy ionizing radiation to induce DNA damage and disrupt cell cycle progression. In the treatment of cancer, RT can be given alone or coupled with chemotherapy or surgery and is aimed at reducing local tumor burden. The primary advantage, however, that RT confers over chemotherapy is the ability to precisely target the tumor and reduce systemic side effects. Epidemiological studies have reported that almost 54% of breast cancer survivors were treated with radiation therapy in 2016 and this is projected to become 60% by 2030 (1). Treatment mode is usually determined by stage and type of cancer, genetic mutations, age, and overall health of patient.

RT can be delivered in several ways; the most commonly used modality is broadly defined as External Beam Radiation Therapy (EBRT), which includes Stereotactic Body Radiation therapy (SBRT) and Stereotactic Radiosurgery (SRS). EBRT most typically uses a linear accelerator to deliver radiation directly into the cancer site in the form of photons. Depending on the location of the tumor, this radiation can be of high or low energy. For instance, high energy EBRT is used in the treatment of head and neck cancer, breast, lung, and eye cancer (2–5) while lower energy photons are used for more superficial cancers such as melanoma (6). Another modality of delivery is brachytherapy, which utilizes a radioactive source placed as close to the tumor as possible and can be given in conjunction with EBRT (5, 7, 8). Some examples of cancers where brachytherapy is frequently administered are cervical, vaginal, and prostate cancer (8–11). Ideally, RT will preferentially or more frequently damage DNA of cancer cells, with less or reparable damage to surrounding healthy cells. Similar to the brachytherapy concept, IntraOperative Radiation Therapy (IORT) constitutes the precise delivery of radiation to the tumor/tumor bed during surgery while minimizing exposure to the surrounding healthy tissues. IORT can be done utilizing electrons, low-kV X-rays, and high dose rate (HDR) brachytherapy. TARGIT, an international randomized clinical trial designed to test the hypothesis that delivering a single dose of targeted IORT in patients eligible for breast conserving surgery (+ EBRT in patients at high risk for local recurrence) is equivalent to a conventional course of post-operative EBRT showed that there was no statistically significant difference between EBRT and the IORT approach with respect to local recurrence-free survival, invasive local recurrence-free survival, mastectomy-free survival, distant disease-free survival or breast cancer mortality (12). In a study looking at brain metastases, retrospective data suggests that IORT is a safe and effective tool in the adjuvant setting following surgical resection of brain metastases; an area that continues to be under debate (13). IORT is currently under investigation in the adjuvant setting following the maximal safe resection of recurrent glioblastoma multiforme (GBM) (NCT04763031, NCT04681677).

Conventional fractionated EBRT was traditionally based off the classical “four R’s” of radiation biology: reassortment, repair, reoxygenation, and repopulation (14), to which radiosensitivity was later added (15). IORT on the other hand is generally performed with either low energy X-rays or electrons; both of which are considered low linear energy transfer (LET) radiation compared to high energy X-rays used in conventional EBRT. Unlike high LET radiation where the linear quadratic model (L-Q) model predicts that radiobiological effectiveness (RBE) should decrease as the dose per fraction increases (16), evidence suggests that this may not be true for low-LET radiation. With a predicted higher RBE, emerging evidence suggests that IORT can be effective by overwhelming the repair system leading to increased genomic instability and thus more cancer cell killing. Additionally, IORT performed during surgery eliminates repopulation of residual tumor cells in the tumor bed, which could theoretically happen during wound healing (17). The ability of IORT to eliminate repopulation could also be attributed to the radiation-induced bystander effect (RIBE) which is thought to be more common with high dose/fraction as is the case with IORT. Abscopal effect in normal non-irradiated cells in the vicinity of tumor could reduce tumor recurrence, modifying the wound microenvironment, and eradicating residual tumor cells when applied immediately after surgical procedure (18).

Additionally, SBRT or SRS is used to deliver very high doses of radiation to the primary sites or metastatic sites in few treatments (1–5), with extraordinary precision made possible by real-time monitoring of the patient under CT scan throughout the duration of therapy. Together they can be combined into a term Stereotactic Ablative Radiotherapy (SABR). Unlike IORT, which arguably does not fit the current L-Q model, current data suggests that this is not the case for SABR, which behaves biologically similar to conventionally fractionated EBRT. However, the higher tumor control that is achievable with SABR when compared to conventional EBRT is attributed to a more geometrically precise technique of dose delivery that allows for prescribing high biological effective doses (BED), which were simply unachievable with conventional dose delivery techniques (19). Additionally, ablative effect on the surrounding tumor endothelium provides additional mechanism of death that is not as prominent in conventionally fractionated EBRT. Emerging data suggest that better tumor control with SABR could also partly be attributable to the abscopal effect brought about by high dose radiation in non-irradiated cells such as enhanced endothelial cell damage and/or enhanced tumor immunity similar to what was suggested in the setting of IORT (20).

## How Does Radiation Work: The Biologic Effects of Radiation

### Effects of Radiation Therapy: DNA Damage

Ionizing radiation introduces energy into molecular structures which then releases electrons creating ions that are capable of breaking covalent bonds. The breakdown of these covalent bonds within DNA produces DNA breaks, including double-stranded breaks. Radiation also leads to the generation of reactive oxygen species (ROS) which oxidize lipids and proteins and are capable of damaging DNA in many ways, including single-strand breaks. This damage leads to cell death and failure of mitosis.

Consequently, highly proliferating cells are most susceptible to damage due to radiation. DNA damage is not an uncommon phenomenon, with as many as 50,000 lesions, or instances of DNA damage, in each cell, every day. Cellular mechanisms of DNA repair are able to fix this continuous damage and maintain functional DNA. Endogenously induced lesions are generally isolated and more evenly distributed throughout DNA. Damage resulting from radiation is far less dispersed. When two or more lesions are found within two helical turns, this is referred to as a clustered damage site, and these are far more difficult to repair than isolated lesions (21, 22). The most highly damaging effect of ionizing radiation is considered to be the double-stranded DNA breaks where both phosphodiester backbones of the two strands of DNA are broken within 10 base pairs (23–28). Double-stranded DNA breaks are likely particularly cytotoxic as they are not regularly induced endogenously (28–30). The linear energy transfer (LET) ratio of the radiation determines the type of damage it induces in the DNA. Particles with a higher LET (e.g., protons, neutrons, alpha particles) results in roughly 90% of the damage occurring in the form of clustered damage sites, while low LET radiation (e.g., gamma rays, x-rays, and electrons) produces roughly 70% of its damage as isolated lesions and the remaining 30% in the form of clustered damage sites (23, 31).

**Figure 1:**
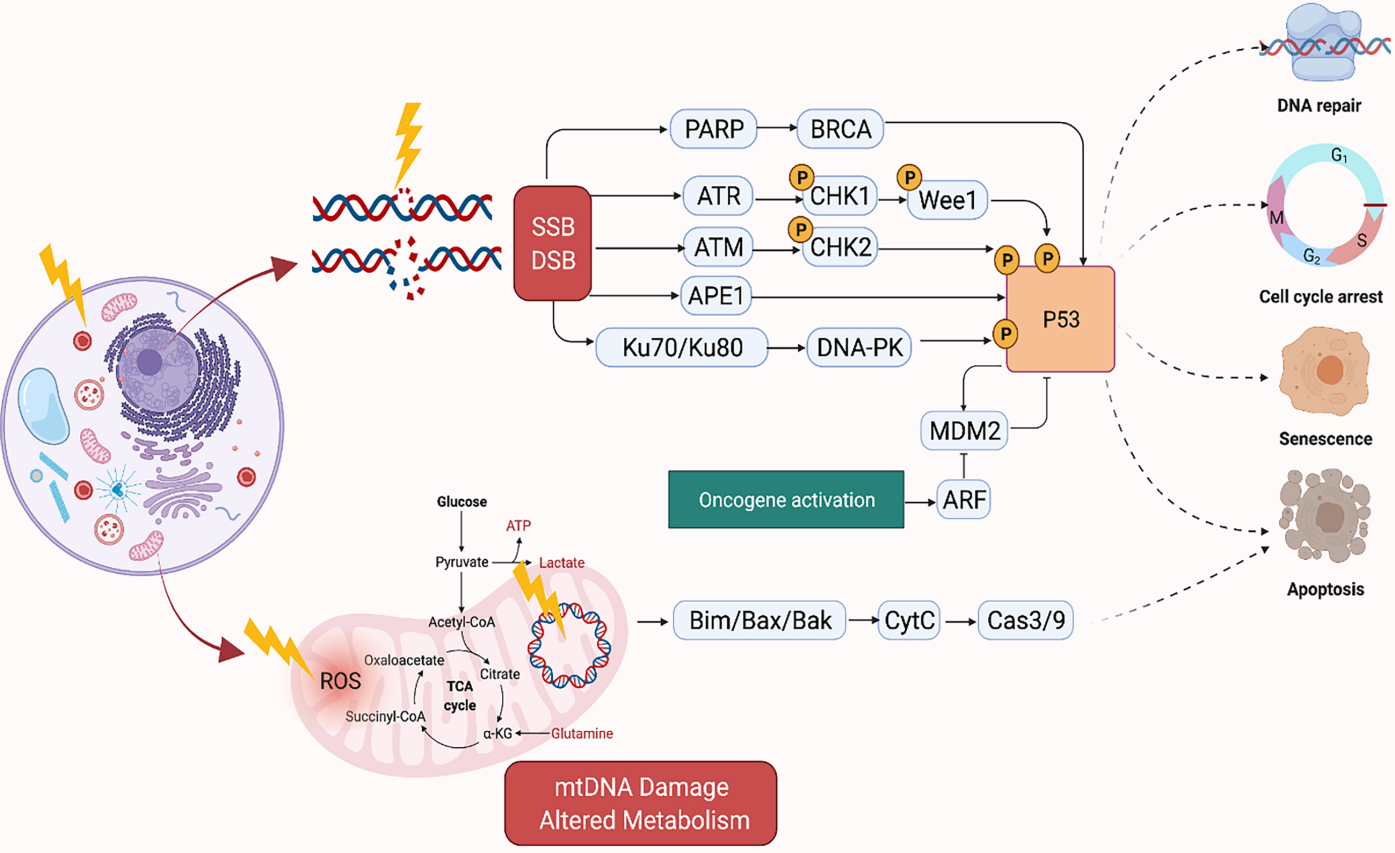
Mechanism of DNA Damage Induced by Ionizing Radiation. Created in BioRender.com.

Radiation kills cancer cells either by damaging the DNA directly or generating excessive ROS which damages the DNA (**Figure 1**). However, cancer cells can become resistant to RT *via* several mechanisms which enhance their DNA repair capacity or suppress the functions of tumor suppressors (32). Therefore, strategies that disrupt the DNA repair machinery or the detection of DNA damage has largely been explored to enhance radiosensitization of tumors. Inhibitors of DNA repair proteins have widely been studied alone or in conjunction with radiotherapy to enhance tumor suppression. For instance, the inhibition of the DNA base excision repair (BER) protein apurinic/apyrimidinic endonuclease, APE1, has been shown to suppress growth of several cancers (33, 34). Similarly, overexpression of APE1 has been linked to radioresistance (35, 36), and suppression has been shown to enhance cancer cells to RT (37). Inhibition of several other DNA repair proteins such as Poly (ADP-ribose) polymerase (PARP) and ataxia telangiectasia mutated (ATM) have demonstrated similar effects (38, 39). The quantity and characteristics of DNA damage are also impacted by the tumor microenvironment, with the oxygen levels of the tumor being of particular importance. Hypoxic tumors do not respond as well to radiation therapy compared to tumors that are well oxygenated. This is because oxygen reacts very quickly with DNA radicals that result from radiation to produce DNA lesions when it is present. Molecules that will react with the DNA radicals can be introduced and function in a similar capacity to oxygen, such as nitroaromatic compounds (e.g., nimorazole, nitrotriazole or sanazole) (40, 41). Nitric oxide is another molecule that is of interest in this regard, though some of its effect may be due to increased oxygen tension of the tumor microenvironment (TME) (42). Due to the potential clinical impact, many preclinical studies have investigated the use of radiosensitizing agents to increase tumor cells’ susceptibility to RT which will be discussed in the sections below (43–46).

### Effects of Radiation Therapy: Cellular Damage

Traditionally, RT has been reported to arrest cancer cell proliferation by inducing DNA damage through stimulation of cell death mechanisms such as apoptosis, necrosis, and senescence. However, radiation can also inhibit cell proliferation by disrupting the neoplastic cells physically through damage to the cell membrane and organelles, and thereby interfering with signal transduction (47–49). Damage to several organelles including the endoplasmic reticulum, ribosome, lysosome, and mitochondria have been implicated in the effects of RT-induced tumor cell death (50–59).

The mitochondria, in particular, is an important target of RT as it regulates cellular respiration and metabolism, and altered metabolism is considered a hallmark of cancer (**Figure 1**) (60). RT-induced damage within the mitochondrial DNA can induce programmed cell death in cancer cells (61). The mitochondrial respiratory chain generates ROS as a byproduct of cellular respiration in normal cells. On the other hand, excess ROS production can potentiate tumor growth. Together, this suggests that cellular response to ROS varies according to levels of ROS generated in the cells. For instance, tumorigenic events such as hypoxia or oncogene activation can induce tumor growth by generating abundant ROS to drive cell cycle progression, metastasis, angiogenesis, etc. However, RT can generate an ROS overload which can arrest the cell cycle and induce apoptosis through mitochondrial collapse in cancer cells (62, 63). For example, FLASH radiation is a novel radiotherapy technology, defined as a single ultra-high dose-rate (≥40 Gy/s) radiotherapy, which unlike conventional dose-rate radiation (described above) leads to strikingly differential responses between healthy and tumor tissues. This differential effect has been attributed to multiple theoretical mechanisms such as distinct mechanisms of DNA damage and the significantly higher ability of FLASH to produce ROS at a rate that can’t be scavenged by tumor cells compared to healthy cells which have a lower oxidant load and higher catalase reduction reserve capacity. More future studies are needed to better understand the mechanism of FLASH and its clinical implications (64).

Several strategies targeting the mitochondria to sensitize cancer cells to RT have been investigated (65–67). The mitochondrial respiratory chain generates ROS as a byproduct of cellular respiration, and RT also generates an ROS overload which can induce apoptosis through mitochondrial collapse in cancer cells (62). LKB1 (also known as serine-threonine kinase 11, STK11) is a tumor suppressor and functions in the AMPK (adenosine monophosphate-activated protein kinase) pathway necessary for cell metabolism, homeostasis, and autophagy (68). In esophageal cancer, overexpressed LKB1 has been reported to confer resistance to radiation therapy, activate autophagy, and inhibit apoptosis (69). One of the metabolic changes that cancer cells initiate during low glucose conditions is the switch from glycolysis to oxidative phosphorylation (OXPHOS) to adjust to fluctuating microenvironmental conditions (70, 71). Irradiated human esophageal adenocarcinoma cells had a higher number of mitochondria with additional mitochondrial mutations compared to their non-irradiated counterparts. Analysis of patient tumors of esophageal adenocarcinoma showed an increase of ATP5B, a marker of OXPHOS, in patients who had poor response to neoadjuvant chemoradiation therapy, suggesting that changes in mitochondrial metabolism can potentially play a role in radioresistance (71).

## Radiogenomics and Rational Design for Radiation-Targeted Therapy Combinations

The combination of radiation therapy and traditional cytotoxic chemotherapy is a clinically well-established approach to improve overall survival of cancer patients (72). However, to date, despite the significant advancements in developing molecularly targeted therapy, little progress has been made in identifying and defining optimal targeted therapy and radiotherapy combinations to improve the efficacy of cancer treatment (73). The rapidly growing arsenal of targeted therapies can be categorized according to their respective effects on one or more of the hallmarks of carcinogenesis which were coined by Hanahan and Weinberg (74, 75). Importantly, the clinical success of these agents was largely based on the identification of predictive biomarkers of response, which enabled the selection of patients and/or tumors that would benefit from these novel agents. This subsequently led to the rise of precision medicine and simultaneously sparked interest in the concept of ‘precision radiation medicine’, yet that concept remains in its infancy.

Precision radiation medicine proposes to leverage genomic information derived from human cancers or preclinical tumor models to identify subsets that are sensitive to specific radiation/drug combinations, radiation alone at tailored doses or predict those at high risk for radiation-related normal tissue side effects (76, 77). As our knowledge of how radiation works evolved over time (as outlined above), several groups have attempted to characterize preclinical models, particularly cell lines to identify genomic signatures that are predictive of radiation sensitivity. The largest effort to date was done by Yard et al., who underwent large-scale profiling of cellular survival after exposure to radiation in a diverse collection of 533 genetically annotated human tumor cell lines and were able to demonstrate the wide range of radiation susceptibility and the novel genetic features driving that diversity (78). Currently, there are several genomic signatures that have been clinically validated for guiding radiation treatment. For example, OncotypeDX^®^, a 21 gene classifier that was initially validated to predict the benefit of adjuvant chemotherapy in hormone receptor positive breast cancer, is currently used to estimate the risk of locoregional recurrence after radiation for invasive breast cancer and therefore guide addition or omission of radiation in the adjuvant setting (79). Similarly, for ductal carcinoma *in situ* (DCIS), DCISionRT^®^ is a multigene assay (80) that has been prospectively validated in 327 patients with DCIS that participated in the E5194 trial (81) to help inform decision-making regarding the addition of radiation in the adjuvant setting in conjunction with clinic-pathologic criteria (82, 83). Decipher^®^ is a 22 gene classifier that was developed as a prognostic tool for men with high-risk prostate cancer and was prospectively validated to guide that addition of post-prostatectomy radiation in that risk group whether in the adjuvant or salvage setting (84, 85). In the 2019, the American Society of Clinical Oncology (ASCO) guideline on molecular markers in localized prostate cancer, only Decipher was recommended to guide the decision between salvage and adjuvant radiation and Decipher^®^ PORTOS was the only predictive signature of radiation response (86). Nonetheless, salvage radiation is generally preferred based on randomized data (87) and so far genetic testing is not part of the standard of care to guide radiation timing until validated in the randomized setting (NCT02783950) (88). In a collaborative novel effort to personalize radiation dose based on genetics and transcend the ‘one size fits all’ paradigm, a novel algorithm that uses genomic adjusted radiation dose (GARD) was proposed to independently quantify differences in clinical outcomes across different cancers that are not attributed to the physical radiation dose alone. This effort aims to guide the integration of genomics into radiation dose decisions (89–92).

While several genomic signatures have been studied in the preclinical setting and a few have been clinically validated to better tailor radiation therapy, limited clinical trials with RT were designed to prospectively test whether specific patient subpopulations with distinct genomic signatures would benefit from radiation or not. For example, HN002 is a phase II study that evaluated radiation dose de-escalation in patients with human papilloma virus (HPV) positive oropharyngeal cancers who are thought to have improved survival outcomes due to impaired DNA repair (93–95). In that study, radiation dose de-escalation was found to be non-inferior to standard dose, which justifies hypothesis testing in the phase III setting. Another eloquent example is in pediatric medulloblastoma where several trials are investigating tailoring radiation dose and technique based on distinct molecular subgroups rather than clinic-pathologic characteristics per say (96). Recently, the ACNS0331 trial demonstrated that reduction of boost volume but not craniospinal radiation dose is safe in average risk medulloblastoma patients and this may occur in a genetic subgroup-dependent manner (97).

The equally important aspect of radiation therapy, which is crucial for an optimal therapeutic ratio, is better understanding and prediction of normal tissue toxicity, particularly late side effects, which are usually irreversible and can severely impact quality of life. While demographic and clinical factors are well-recognized culprits of late tissue toxicity, the evolving field of radiogenomics proposed genetic factors as key players as well. Kerns et al. proposed two arching goals for the field: first, identifying key molecular pathways that can predict radiation-induced normal tissue toxicity and second, developing an assay to identify the patients who are more likely to develop late tissue toxicities and therefore require tailored treatment (98). Several genome-wide association studies have identified associations between specific single nucleotide polymorphisms (SNPs) and radiation toxicity (99–101). The REQUITE international prospective toxicity profiling effort, initiated by The Radiogenomics Consortium, represents the largest study to date in that regard and has led to the creation of a centralized database of relevant clinical information including treatment, dosimetry, toxicity, and genome-wide SNP genotyping data in an effort to prospectively validate these findings for clinical use (102, 103).

Despite the efforts outlined above, the radiation oncology field significantly lags behind in designing clinical trials that are poised to prospectively test whether specific combinations of radiation and targeted therapy can particularly benefit a genomically distinct patient population. To that end, several collaborative efforts aimed to outline guidelines to usher the field toward optimizing the clinical development of novel drug-radiotherapy combinations. Two key points were proposed: 1) reconsidering novel endpoints in clinical trial design such as local control, organ preservation, and patient reported outcomes, and 2) prioritizing the development of promising therapeutics that target relevant pathways to radiation such as DNA repair inhibitors and immunotherapies (104–106).

Traditional radiosensitizing agents (such as cisplatin and 5-fluorouracil) typically exert their effect by augmenting DNA damage (72). As large genomic studies continue to unravel the landscape of DNA repair pathway deficiencies across different tumor types, it will be critical to propose novel rationally designed combinations of radiation and targeted therapy that fit specific genomic contexts (77). PARP1, WEE1, DNA-PK, ATM, ATR, and CHK1 are among the most critical mediators of DNA damage response (DDR) (**Figure 1**). DDR inhibitors (such as PARP inhibitors) were initially developed as monotherapy to target DDR defects that are present in tumor cells, but not in normal cells. This selectivity gave rise to the concept of synthetic lethality (107). Theoretically radiation is an attractive DNA-damaging agent that can be combined with novel DDR inhibitors to promote cell-selective radio-sensitization by three mechanisms: firstly, by increasing the amount of DNA damage to levels that induce apoptosis or cell death mechanisms rather than DNA repair or cell cycle arrest, secondly by exploiting synthetic lethality, and thirdly, by augmenting DNA damage and thus increasing the tumor mutation burden which in turn enhances tumor antigenicity and thus T-cell mediated killing (108).

**Figure 2:**
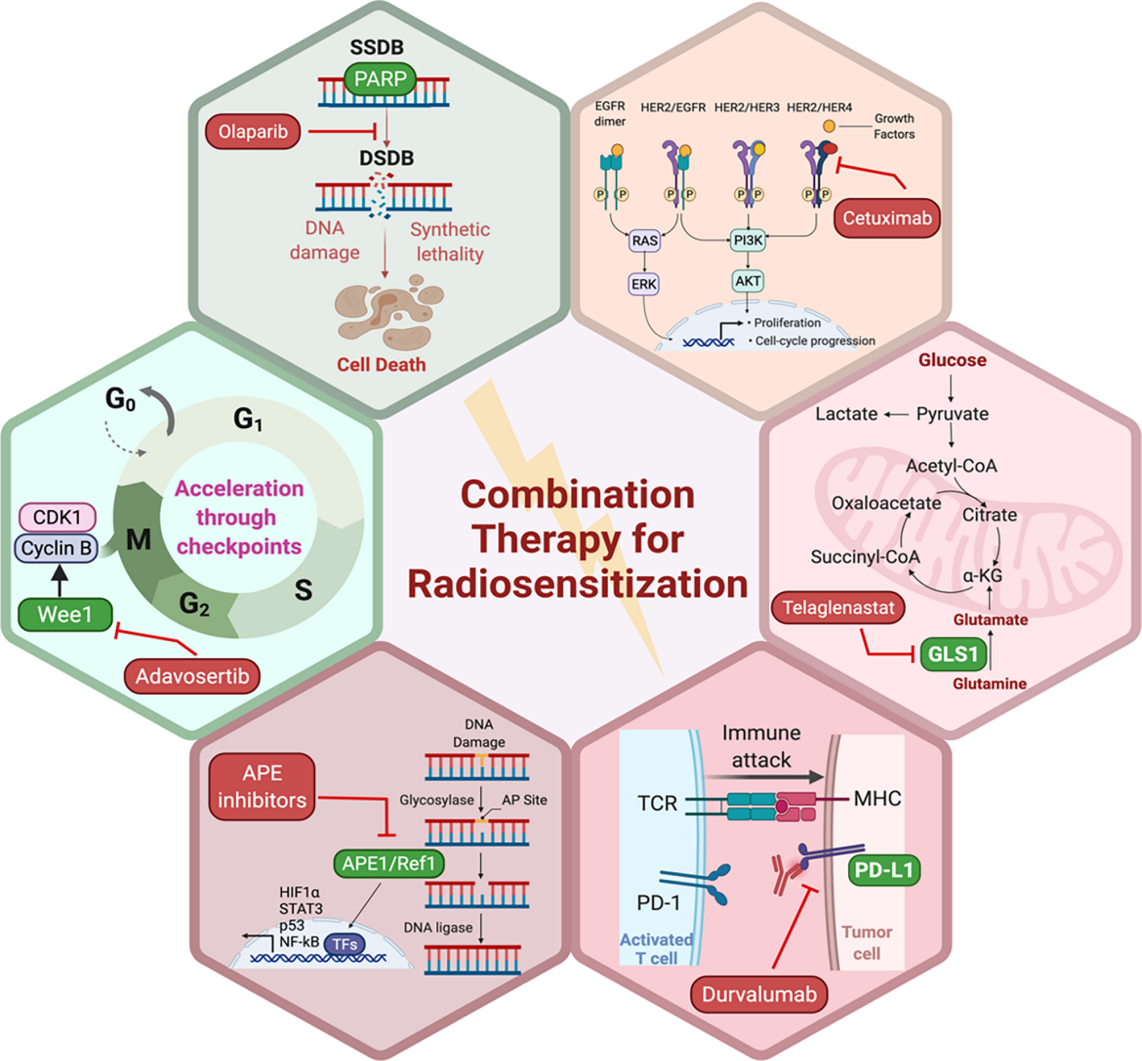
Potential pathways and representative small molecule inhibitors of the key proteins in those pathways with potential to enhance the sensitivity of tumor cells to RT. Created in BioRender.com.

Preclinical evidence suggests that DDR inhibitors can act as potent radiosensitizers and potentially have greater cytotoxic effects in cancer cells compared to normal cells (**Figure 2**). This also brought about the idea of synthetic lethality in which cancer cells, unlike their healthy counterparts, carry DNA repair defects, making them particularly vulnerable to DDR inhibitors, especially when simultaneously targeted with a DNA damaging agent such as radiation (109, 110). For example, PARP inhibitors have been shown to be potent radiosensitizers, irrespective of the tumor’s homologous recombination (HR) status (111), albeit at lower doses in HR-deficient tumors (112). Similarly, Adavosertib, a WEE1 inhibitor is also an effective radiosensitizer (113, 114). Inhibition of WEE1 abrogates the G2/M checkpoint which is crucial for P53 mutant cancer cells, which also lack the G1 checkpoint. Therefore, WEE1 inhibition represents another form of tumor-selective radiosensitization (115). Induction of replication stress is another appealing mechanism that can selectively enhance radiation sensitivity in cancer cells particularly in the context of cMyc and KRAS mutations (116, 117). Several DDR inhibitors including PARP, WEE1, and ATR inhibitors have been implicated in the induction of replication stress either as monotherapy or in combination with other DDR inhibitors together with RT (118). Several clinical trials are currently testing the premise of combining radiation with DDR inhibitors in various disease sites.

In the era of immunotherapy, modulation of the host and the tumor microenvironment holds a lot of promise when combined with radiation as demonstrated in a plethora of eloquent preclinical studies. Radiation and immunotherapy agents are thought to interact through five distinct mechanisms based of the modified Steel hypothesis (119): (1) spatial cooperation, (2) temporal modulation, (3) biological cooperation, (4) cytotoxic enhancement, and (5) normal tissue protection (120). Radiation has immunostimulatory and immunosuppressive effects. Radiation can induce immunogenic cell death and increase expression of tumor specific antigens and thus sensitize tumors to the effects of immunotherapy (121, 122). In the preclinical setting, Twyman-Saint Victor et al. demonstrated synergy between radiation therapy and combined anti-PD-1/PD-L1 and anti-CTLA4 blockade. In this study, the combination led to an increased response within the tumor as the radiation induced the diversification of the T-cell repertoire in tumor-associated lymphocytes and the immune checkpoint inhibitors inhibited T-regulatory cells (Tregs), which resulted in an increase in the CD8/Treg ratio and subsequently led to improved outcomes compared to either modality alone in a variety of tumor models (123). The abscopal effect of radiation refers to another form of RT-immunotherapy synergy where anecdotal studies (mostly in patients with melanoma) have shown tumor response in non-irradiated lesions presumably due to an incited systemic immune response resulting from local radiation treatment (20, 124–127). Conversely, radiation can promote tumor infiltration by suppressive regulatory T cells, inhibitory macrophage and myeloid-derived suppressor cell lineages (128, 129), therefore combination with immunotherapy in that context is crucial to maintain the anticipated cytotoxic effect of RT. The optimal dose, fractionation, volume, and sequencing of RT with immunotherapy remain to be elucidated to strike the balance between the immunostimulatory and immunosuppressive effects of radiation and to fulfill the modified Steel criteria (76, 77, 120).

Thus far, the failure to predict treatment efficacy using genetic variables represents one of the most significant obstacles to the personalization of radiation-based treatment regimens. The potential success of radiosensitizing-targeted therapy is contingent upon our better understanding of radiogenomics, which pertain to defining biomarkers of response and genetic determinants of late tissue toxicity (106, 130, 131). Moving forward, two key concepts need to be considered in order to facilitate rational design of novel radiation-targeted therapy combinations that are effective: redefining end points of interest and efficacy and identifying and validating biomarkers that can enable the early identification of ineffective or toxic compounds. These two key concepts will require the optimization of preclinical models that can accurately recapitulate the complexity of human tumors and thus faithfully predict promising combinations and subsequently re-thinking clinical trial design in a way that is relevant to radiation and its paradigm.

## Building Predictive Experimental Models in the Validation of Combination Therapy That Includes Radiation

For a small molecule to be maximally effective as radiosensitizer, it must be highly specific and directly toxic to the tumor. Tumor cells depend more heavily on certain signaling pathways over normal tissues, therefore combination of RT with small molecule inhibitors of these pathways offers an alternative strategy to chemoradiation that is potentially less toxic to surrounding healthy tissues. A general limitation to this is the lack of preclinical models that mimic the human cancer to a molecular level which provides information regarding predictive biomarkers that differentiate between radioresistance and radiosensitivity.

Preclinical models for studying cancer radiogenomics as well as cancer efficacy studies require recapitulation of human cancer on an anatomical and histological level in a manner that closely mimics the human tumor characteristics. The driver or passenger mutations, microenvironment, hypoxia, angiogenesis, immune components, and therapeutic response are all important factors to consider. Therefore, several approaches are being used to build multi-cellular *in vitro* models as well as *in vivo* models with appropriate genetic manipulations to capture the aforementioned characteristics in response to RT. Methods include genetic knockdown, knock-in, activation, tissue-specific expression, inducible expression, and sequential expression in traditional cell culture, 3-dimensional (3D), organoid, and xenograft models. My laboratory has focused on generating 3D mono- and co-cultures using various cancers such as pancreatic, colon, and bladder (132–135). The use of both tumor cells and CAFs with distinct fluorescent markers allows us to monitor the effects of both cell populations following selective pathway inhibition. For example, we demonstrated the enhancement of tumor cell killing with dual inhibition of APE1/Ref-1 as well as CA9 (carbonic anhydrase 9), a HIF-1α target. Through blocking the full activation of HIF-1α through APE1/Ref-1 and the cells ability to respond to changes in pH through CA9, the spheroid growth was dramatically reduced (135). This model is now being interrogated to understand the effects of RT on growth of the spheroids and the impact on the cells of the TME as well as RT in combination with targeted agents that would impact hypoxia as well as metabolic signaling.

*In vitro* models often use a panel of radiosensitive and radioresistant cell lines and compare the effects of select small molecule inhibitors or the effects of knocking down potentially important signaling molecules. Other approaches include generation of radioresistant lines and determining which molecular factors play a role in their resistance. 3D models can aid in recapitulating the cell-cell interactions within tumor and stroma, cytokine signaling, hypoxia response, and combination therapy involving RT and allow us to quantitate the effects on the tumor as well as cells from the TME such as CAFs (136–138). A study comparing radiosensitive and radioresistant non-small cell lung cancer (NSCLC) demonstrated that pathways previously implicated including DNA repair, apoptosis, and NFκB activation in NSCLC were involved in the cellular response to RT (54). Prostate cancer cell lines and the transgenic mouse model TRAMP (Transgenic adenocarcinoma of mouse prostate) used natural product, Nexrutine (Nx), to sensitize the prostate cancer cells to RT both *in vivo* and *in vitro*. Downregulation of ribosomal and cell cycle proteins as well as HIF-1α were implicated in the sensitization of the tumors to Nx (56). These are just two examples of preclinical studies that utilize various models to test the radioresistance and sensitivity of various cancer types. The predictability of the model and the complexity of the 3D or monolayer system in response to RT will enable the preclinical studies to have a greater impact on the rationale design of combination therapy which will ultimately lead to translational impact.

## Rational Combinations of Radiation and Targeted Therapy in the Preclinical Setting

PARP proteins are involved in DDR and inhibitors of PARP have been widely studied for radiosensitization both preclinically and in the clinic (discussed below and **Figure 2**). Currently, there are four PARP inhibitors in the clinic: Olaparib, Rucaparib, Niraparib, and Talazoparib (**Table 1**). The efficacy of this combination therapy has also been studied in preclinical models of human non-small cell lung cancer (NSCLC): Calu-3 and Calu-6 cell lines. Even though both cell lines exhibited increased radiosensitization following Olaparib treatment *in vitro*, only xenografts of Clau-6 showed increased response to combination RT *in vivo*. Difference in response between Clau-3 and Clau-6 were most likely due to microenvironmental factors that contributed to the sensitivity of cells, indicating that preclinical modeling must be approached unbiased and carefully with the appropriate TME (139). Talazoparib and Niraparib have also been studied for their sensitizing effects. Primary melanoma cultures treated with combination therapy of Talazoparib, Niraparib and radiation, demonstrate that both PARP inhibitors sensitize melanoma cells to IR (162). A short-term phase 1 clinical trial looking at the efficacy of combination therapy of radiation and Olaparib has determined the safety of the combination regimen in doses up to 200 mg/day without any side effects (163).

**Table 1:** List of radiosensitizers, respective mechanism of actions and preclinical models used to study them.

Radiosensitizer	Mechanism	Cell models studied	References
Olaparib	Blocks DNA repair by inhibiting PARP	Breast cancer: MCF-7, MDA-MB-231, MDA-MB-231, T47D, BT-549, HCC-1954 NSCLC: Clau-3, Clau-6	(139, 140)
Rucaparib	Blocks DNA repair by inhibiting PARP	Cervical cancer: HeLa Prostate cancer: PC3, LNCaP, DU145, VCaP Neuroblastoma SK-N-BE(2c), UVW	(141–143)
Cetuximab	Inhibits epidermal growth factor (EGF) from binding to its receptor	HNSCC: HN30, HPV-negative HTB-43, UM-SCC1, UM-SCC2, UM-SCC6, HPV-positive UM-SCC47, UPCI : SCC090 cells	(144, 145)
Telaglenastat	Interferes with mitochondrial metabolism by inhibiting the conversion of glutamine into glutamate	HNSCC: FaDu, HN5, CAL-27 Lung Cancer: H460, A427, A549	(146, 147)
Tirapazamine	Selective for hypoxic cells; Generates reactive oxygen species which cause DNA damage	Human Nasopharyngeal Carcinoma: HNE-1 Cervical cancer: HeLa	(148, 149)
Everolimus	Inhibits mTOR kinase	NSCLC: NCI-H460, NCI-H661 Glioblastoma: GS-2	(150, 151)
Nimorazole	Generates reactive oxygen species which cause DNA damage	HNSC: HPV-negative FaDu, UTSCC5, UTSCC33 and HPV positive: UMSCC47, UDSCC2 UPCISCC90	(152)
Trametinib	Inhibits MEK	NSCLC: A549, H460 Melanoma: A375, D04, WM1631, WM1791c	(153, 154)
Adavosertib	Inhibits Wee1 and impairs the G2 DNA damage checkpoint	Esophageal Cancer: OE33, FLO1	(155)
Peposertib	Inhibits DNA-PK and impairs DNA repair	Leukemia: Molm-13, Molt-4 HNSCC: FaDu Colon Cancer: HCT116	(156, 157)
Silver NP	Deposit high levels of energy in cells when exposed to ionizing radiation; ROS generation and DNA damage	Glioma: C6 Colon cancer: HCT116, HT29	(158, 159)
Gold NP	Deposit high levels of energy in cells when exposed to ionizing radiation; ROS generation and DNA damage	Breast Cancer: SK-BR-3	(160)
Bismuth NP	Not fully understood; Possibly by depositing high levels of energy in cells when exposed to ionizing radiation; ROS generation and DNA damage	Breast cancer: MCF-7, 4T1	(161)

Apurinic/apyrimidinic endonuclease 1/Redox factor-1 (APE1/Ref-1) possesses multiple functions that could affect the cellular response to RT (**Figure 2**). APE1/Ref-1 is key in the base excision repair (BER) pathway of DNA lesions, acting as the major AP endonuclease in both the nucleus and mitochondria and in eukaryotic transcriptional regulation of gene expression as a reduction-oxidation (redox) factor (164–166). APE1 contributes to the repair of ionizing radiation through its ability to repair a 3’-phosphoglycolate end within a DNA strand break that is generated following ionizing radiation (IR) (167). A decrease in expression of APE1/Ref-1 in cancer cells results in apoptosis, cell cycle arrest, a decrease in proliferative capacity, a blockade of mitochondrial metabolism, and sensitization to various anti-cancer agents including RT (166, 168–170). Biochemical studies using oligonucleotides with clustered damage sites as would be encountered in a cell following RT demonstrate that APE1/Ref-1 can repair these types of DNA lesions (171). An inhibitor of the DNA repair activity of APE1/Ref-1 has been difficult to identify and develop preclinically, therefore two recent studies in pediatric and adult brain tumors utilized nanoparticle delivery of APE1/Ref-1 siRNA to achieve sensitivity to RT (168, 169). One of APE1/Ref-1’s interacting protein partners is nucleophosmin 1 (NPM1) and perturbation of the APE1/Ref-1 – NPM1 interaction can lead to decreased DNA repair activity of APE1/Ref-1 and increase in sensitivity to chemotherapeutic agents such as bleomycin (172, 173). Recently in NSCLC cells, radiosensitizing agent YTR107 was shown to bind to NPM1, disrupt RAD51 foci formation, and synergize with PARP inhibition (174). These findings highlight the complex interplay between radiation-induced DNA damage and repair and the potential proteins that can be exploited as drug targets to sensitize cancer cells to RT. Due to APE1/Ref-1’s role in the repair of DNA lesions induced by RT, the blockade of APE1/Ref-1 DNA repair activity could be highly effective in combination with RT. The caveat of course would be toxicity to normal tissues, and therefore development of tumor targeting strategies would be of paramount importance.

In addition to DNA repair activity, APE1/Ref-1 also plays an important role in signaling within the tumor and TME through the transcription factors (TFs) it regulates, and many of these TFs also play a role in inflammation (166). Functioning as a redox factor, APE1/Ref-1 stimulates the DNA binding activity of TFs by reducing cysteine residues within the TF (175). APE1/Ref-1 activates TFs including HIF1a, STAT3, p53, NF-kB and others that directly govern critical cellular functions, including hypoxia, DNA repair, inflammation, and angiogenesis (166). Cells, both tumor and normal, possess reduction-oxidation systems such as NRF2, thioredoxin, peroxiredoxins, and glutathione. In contrast, APE1/Ref-1 functions as a signaling molecule rather than a general redox system (176, 177). Our team has extensively characterized APE1/Ref-1 redox signaling inhibitors in several indications including cancer as well as chemotherapy- or IR-induced neuropathy (165, 178, 179). Vasko et al. demonstrated that the DNA repair function of APE1/Ref-1 was protective against the neurotoxicity induced by IR and APE1/Ref-1 redox inhibitor, APX3330 could protect dorsal root ganglia against IR-induced cytotoxicity (179). Blockade of APE1/Ref-1’s redox activity could also sensitize radioresistant cancer cells or remodel the TME to affect the tumor’s response to RT as HIF, STAT3, NF-kB, and others have been strongly implicated in the cellular response to RT (180–183).

Finally, inhibition of DDR signals by enhancing p53 function has also proven to be effective for radiosensitization in preclinical models. Several strategies employed for this revolve around suppressing the functions of proteins that inhibit p53. For instance, mouse double minute 2 homolog (MDM2) inhibitors have widely been studied for combination radiotherapy in several different cancers which enhance anti-tumor effects *in vitro* and *in vivo* (184–188).

Moving on from DNA damage, the traditional culprit in radiation medicine, now significant interest exists in developing radiosensitizers that more selectively radiosensitize tumors, but not normal tissues, by targeting signal transduction pathways that are more commonly activated in tumors, such as the EGFR pathway (189, 190). Growth factors are essential for cancer cell proliferation and inhibition of apoptosis, and therefore, can contribute to radioresistance *via* several mechanisms, including activating proteins or pathways involved in repairing radiation-induced DNA damage (191, 192). Preclinical evidence has supported a radiosensitizing role for EGFR inhibition (193) and indeed, the addition of cetuximab to RT in patients with head and neck squamous cell cancer was shown to improve tumor control and overall survival compared with radiation alone (189). However, understanding the impact of the spectrum of EGFR alterations on radiosensitivity remains to be understood (194, 195). Similarly, the blockage of ERBB2 (human epidermal growth factor receptor2 [HER2]), which is commonly amplified in a subset of breast cancer (196), can reverse ERBB2-mediated radioresistance (197). These findings were translatable into the clinic which was evident from a recent analysis of the HERA trial which demonstrated the potential of combining radiotherapy with trastuzumab in reducing loco-regional recurrence rates in breast cancer patients with 1 to 3 positive lymph nodes (198). In the prostate cancer field, the combination of androgen deprivation therapy (ADT) and radiation in patients with intermediate and high risk prostate cancer is a well-established approach to prolonging survival in that subset of patients (199). Despite being one of the earliest examples of combining radiation with targeted therapy, the mechanism of synergy between ADT and radiation remains controversial. Initially much of the benefit was thought to be derived by the orchestrated effect of radiation controlling disease locally in the prostate and ADT treating micrometastatic disease elsewhere (200). Newer preclinical data suggests that ADT has direct effects in the prostate that result in radiosensitization *via* several mechanisms including relieving hypoxia (201), suppressing DNA repair (202) and deactivating androgen receptor (AR). The blockade of AR signaling is thought to regulate the transcription of DNA repair genes and thus mediate radioresistance (203). The modulation of several other oncogenic pathways could provide another approach to enhance radiation sensitivity such as intracellular signaling (i.e. PI3K/AKT pathway) (204) and tumor-associated epigenetic changes (205).

As mentioned previously, the rationale for targeting tumor metabolism to sensitize cancer cells to RT is well-established. Mitochondrial metabolism is crucial to cancer cell survival and RT-induced mitochondrial DNA damage as well as excess ROS generation provides an attractive target to suppress cancer cell proliferation and induce apoptosis (146, 147, 206). Glutamine metabolism facilitates cancer cell survival, and breakdown of glutamine is mediated by glutaminases, making them the focus for development of small molecule inhibitors. Indeed, preclinical data supports the combination of the glutaminase inhibitor, Telaglenastat (CB-839), in radiosensitization of cancer cells (**Figure 2**). Telaglenastat suppresses cancer cell proliferation alone and in combination with 5-FU or EGFR in several colorectal and lung cell lines (207, 208). Combination therapy of radiation and Telaglenastat diminishes cancer progression in cell culture and mouse models of head and neck squamous cell carcinoma. Clonogenic cell survival assays with FaDu (pharynx), HN5 (tongue), and CAL-27 (tongue) cell lines treated with radiation and Telaglenastat demonstrated significantly diminished proliferation compared to radiation or Telaglenastat treatment alone. These findings were confirmed using xenograft models in which combination therapy was superior to monotherapy (147). Similar results have been reported in lung cancer radiosensitization where treatment with Telaglenastat increased efficacy of RT by 30% in multiple cell lines and in H460-derived tumor xenografts (146).

Tumor hypoxia is another well-established mediator of radioresistance (209) and typically indicative of aggressive and treatment-resistant disease. Targeting tumor hypoxia by traditional cytotoxic chemotherapy has served as a cornerstone for concurrent chemoradiation regimens for decades. However, the validation of biomarkers of tumor hypoxia in patients that could guide the implementation of novel rationally designed combinations of radiation and hypoxia-targeting agents remains underexplored (105). Historically, several methods have been investigated in order to override hypoxia-mediated radioresistance. Such methods included: hyperbaric oxygen (210), oxygen mimetics which belong to the nitroimidazole class of agents (211), and hypoxia activated cytotoxic prodrugs such as tirapazamine (212). More recently, with the advent of the concept of normalizing tumor blood flow using anti-angiogenic therapy (AAT), several studies proposed RT-AAT combinations to alter oxygenation and improve therapeutic response. In xenograft mouse models, PI3K targeted inhibition led to improved tumor local control following radiation, which was associated with normalization of vasculature and increasing intrinsic radiosensitivity (213). In patients with NSCLC, PI3K inhibition led to reduction in tumor hypoxia as measured by FMISO PET in patients and was well tolerated in combination with palliative thoracic radiation (214). In GBM where angiogenesis is thought to be the hallmark of pathogenesis and VEGF its main driver (215), combining VEGF/EGFR with RT has been shown to halt the growth of glioma cells preclinically (216) and to have a significant synergistic anti-tumor effect with RT (217, 218).

The role of the tumor microenvironment on response to RT alone and in combination with chemotherapy or targeted agents is an important and understudied area. Stromal normalization is one approach to modulating the tumor microenvironment and reducing tumor hypoxia particularly with respect to radiation. Cancer-associated fibroblasts (CAFs) are naturally radioresistant, and data suggests that radiation can induce their pro-tumorigenic capabilities. However, the concept of combining RT with CAF targeting has not been investigated to date (219). Alternatively, another novel paradigm of targeting tumor hypoxia is the modulation of the tumor microenvironment by altering tumor metabolism through the inhibition of oxidative phosphorylation and thus decreasing tumor oxygen consumption rate and relieving hypoxia (220). Atovaquone, an FDA approved anti-malarial that functions through inhibition of mitochondrial complex III has been shown in pre-clinical models to alleviate tumor hypoxia and in turn results in tumor radiosensitization (221).

Finally, owing to rapid advances in nanotechnology, nanomaterials have attracted particular attention to enhance the anticancer efficacy of radiotherapy (158, 161, 222, 223). Nanoparticle delivery enhances tumor targeting while simultaneously improving effectiveness of radiotherapy by increasing local deposition of ionizing radiation dose or by augmenting production of ROS, DNA damage and cell cycle arrest (224). Silver nanoparticles were reported to sensitize both hypoxic and normoxic glioma U251 cells and C6 cells to radiotherapy (222). In additional studies, silver nanoparticles surface modified with polyethyleneglycol (PEG) and aptamer improved nanoparticle penetration and targeting in 3D glioma models, and conjugation with PEG/aptamer further enhanced radiosensitization in C6 xenograft models as well (158). The development of theragnostics further expand the scope of nanoparticles for multifunctional use (161). For instance, PEG conjugated bismuth gadolinium oxide nanoparticles (BiGdO3) not only sensitized breast cancer MCF-7 and 4T1 lines and 4T1 xenograft models to radiation, but the bismuth and gadolinium also allowed for MRI and CT imaging (161).

Even with the multitude of preclinical studies looking at combining RT with targeted therapy, chemotherapy, or immunotherapy, there are still very few examples of combinations that have translated into success clinically. We will now highlight some examples as well as future directions (**Table 1** and **Figure 2**).

## Radiation-Targeted Therapy Combinations in the Clinic: Stories of Success and Failure

A large body of preclinical evidence exists to support novel radiation-targeted therapy combinations. However, to date the EGFR inhibitor cetuximab remains to be the only molecular targeted agent approved by the U.S. Food and Drug Administration (FDA) for use with radiation therapy in head and neck cancer (189). Interestingly however the equivalence of cetuximab and cisplatin as radiosensitizers in head and neck cancer has been a crucial point of contention in the field. A small randomized trial by Margini et al. suggested that cetuximab was inferior to cisplatin when combined with radiation in patients with locoregionally advanced head and neck cancer (225). Two recent large, randomized trials have provided more conclusive evidence that cetuximab is indeed inferior. In the De-ESCALaTE Human Papilloma Virus (HPV trial), patients with low-risk HPV-positive oropharyngeal cancer had higher rates of local recurrence and lower overall survival when treated with cetuximab-RT compared to when treated with cisplatin-RT (226). That was also the case in the RTOG 1016 trial (227).

Although cetuximab was relatively successful as a radiosensitizer in the setting of head and neck cancer, it failed to show promising results in other cancers where EGFR signaling is relevant (**Figure 2**) (228–230). There is also a multitude of phase I/II data that demonstrated similarly disappointing results for other EGFR inhibitors. For example, EGFR is amplified in around 40% of GBM cases and its overexpression is associated with poor prognosis (231–233). Three phase II studies have examined the role of erlotinib, an oral tyrosine kinase inhibitor of the human EGF receptor that is FDA approved for the treatment of non–small cell lung and pancreatic cancers, given concurrently with RT plus temozolomide and have demonstrated widely contrasting results with respect to survival and toxicity. The overall trend however pointed towards increased toxicity with no substantial survival benefit. Phase I and II clinical trials have also been developed to study the combination of RT with erlotinib in pancreatic cancer in both the adjuvant and unresectable, locally advanced settings. Although toxicity profile was acceptable, only modest increases in efficacy have been observed (234–238). Alternative strategies for EGFR targeting have also been attempted in the early clinical settings. For instance, m-TOR targeting which is downstream of the EGFR/PI3K pathway have been trialed in the GBM setting. Two multi-institutional phase II studies have investigated the use of m-TOR inhibitor, Everolimus, in combination with standard RT plus TMZ, The North Central Cancer Treatment Group (NCCTG) N057K trial (239) and The Radiation Therapy Oncology Group (RTOG) 0913 trial (240). Despite having distinct designs, both trials showed no improvement in survival and increased toxicity. The rationale for the combination of EGFR inhibitors with RT is mainly based on the role of EGFR in driving the disease rather than on how the two modalities might work together to kill the tumor. Perhaps in future studies, combinations of RT with targeted agents need to be more rationally designed in order to see greater success clinically.

Another targeted radiosensitizer that has been relatively successful in the clinical setting is nimorazole. Nimorazole is a targeted radiosensitizer which selectively targets hypoxic tumor cells and has been shown in a phase III trial to significantly improve locoregional control by 16% in patients with cancer of the supraglottic larynx and pharynx when combined with radiation compared to radiation alone (241). However, nimorazole is currently only used in Denmark and has failed to become adopted as standard of care in the United States and elsewhere (242). In order to overcome hypoxia to sensitize tumors to radiation, Accelerated Radiation, Carbogen, and Nicotinamide, also known as the ARCON regimen, has demonstrated promising locoregional control rates and yet toxicity in a two large phase II studies in patients with head and neck cancer (243) and bladder cancer, respectively (244). This led to the phase III BCON trial which showed improved locoregional control and overall survival in bladder cancer patients who were treated using that regimen compared to patients treated with conventionally fractionated radiation alone (245). However, in a phase III study testing this regimen in laryngeal cancer patients, there was no significant improvement in either local control nor organ preservation rates in ARCON treated patients albeit with benefit in patients with hypoxic tumors (246). Taken together, this regimen has not been widely adopted due to practical difficulties in delivering this regimen, proper patient selection due difficulties in accurately determining highly hypoxic tumors, and inconclusive results from phase III data (247). Tirapazamine, the most clinically developed drug among hypoxia-activated cytotoxic prodrugs, which represent another class of hypoxia-targeted radiosensitizers (212), have failed in phase III trials to demonstrate improved outcomes when combined with chemoradiation compared to conventional chemoradiation alone in both cervical (248) and head and neck cancers (249). Similarly, VEGF targeting which theoretically represents another attractive way of normalizing tumor vasculature and overcoming hypoxia, failed to improve OS in GBM patients where VEGF targeting was particularly alluring given its centrality to the disease pathogenesis (250–252). Interestingly however, another study showed that GBM patients that have increased tumor oxygenation following anti-angiogenic therapy when combined with conventional chemoradiation live significantly longer (253). Alternatively, targeting the stroma has been clinically attempted for radiosensitization with the goal of modulating RT-induced inflammatory responses (247). Recently, a phase II trial in patients with locally advanced pancreatic cancer has shown that addition of losartan to chemoradiation enhanced tumor shrinkage and enabled more margin negative resections likely due to interfering with TGF-β signaling in CAFs which are characteristic of the desmoplastic tumor microenvironment in pancreatic cancer (254).

Predictive biomarkers of response, which served as the premise of the systemic targeted therapy revolution, are needed in the radiation oncology field to improve trial design and success rates. To that goal, several early-stage clinical trials are currently underway; testing radiation resistance pathways that have been validated in the preclinical setting. For example, KRas, a proto-oncogene that is frequently mutated in a wide range of cancers (255) is a well-known driver of resistance to cancer therapy including radiation (256–258). Several exploratory clinical trials have demonstrated a link between KRas mutation status and decreased likelihood of locoregional control following radiation treatment (259–261). Midostaurin, a multikinase inhibitor that is FDA approved for treatment of FLT3 mutant acute myeloid leukemia (262) is currently being tested in phase Ib trial to be given concurrently with conventional chemoradiation in rectal cancer patients (263). This was based on an *in vitro* screen of 32 cell lines that represented lung, colorectal, head and neck, and genitourinary cell lines and identified Midostaurin as a potential radiosensitizer for KRas mutant cancers (264). Trametinib, a MEK inhibitor that is FDA approved for treatment of metastatic melanoma, is also being tested in a phase I trial in combination with chemoradiation for locally advanced KRas mutant NSCLC (265). Importantly, KRas has been so far inaccessible for direct inhibition until the recent FDA approval of sotorasib for the management of KRas mutated NSCLC based of the CodeBreaK 100 trial (266). It will be interesting to see how this could change the landscape of radiosensitization in the setting of KRas mutated cancer in the near future.

As discussed previously, DNA damage response is central to radiation response. However, so far there are many perceived challenges to clinically implementing this combination such as optimal sequencing, ideal genetic background, and importantly therapeutic window to avoid increased toxicity (267). There are numerous ongoing phase I/II trials combining radiation or conventional chemoradiation with novel targeted DDR inhibitors. Among DDR inhibitors, PARP inhibitors are the most clinically developed followed by WEE1 inhibitor, Adavosertib (**Figure 2**). In inflammatory or locally recurrent breast cancer, a phase I multicenter study evaluated veliparib, a PARP inhibitor, and concurrent RT for 30 patients. The study showed overall acceptable toxicity with only five (16.7%) patients experiencing a dose limiting toxicity (DLT) within 10 weeks from RT initiation. Although severe acute toxicity did not exceed 30% at even the highest dose, nearly half of the surviving patients demonstrated G3 adverse events at 3 years. Of the 30 patients, 15 experienced disease control failures during the 3 years of follow-up and 13 died which highlights the importance of long-term monitoring of toxicity in trials of radiosensitizing agents (268). A phase II trial comparing radiation with or without Olaparib in patients with inflammatory breast cancer, which is known to be particularly aggressive with dismal prognosis (269), is currently recruiting (NCT03598257). In pancreatic cancer, if the patient is homology recombination repair deficient (HRD), this may render the tumor particularly vulnerable to PARPi (270). Velaparib concurrent with chemo-RT was tested in a phase I study of 30 patients with locally advanced disease. Sixteen DLTs were detected in 12 patients (40%). Interestingly, median OS for DDR pathway gene-altered- and DDR-intact patients was 19 and 14 months, respectively. The most commonly mutated DDR gene was ARID1A (n = 4). Loss of ARID1A impairs both checkpoint activation and the repair of DSBs, which sensitizes cells to DSB-inducing treatments such as RT and PARP inhibitors (271). PARP inhibitors are also being tested in conjunction with other forms of targeted therapy such as EGFR inhibitors. A recent phase I study showed that Olaparib may be safely combined with concurrent cetuximab and radiation for patients with locally advanced head and neck squamous cell carcinoma who have a long smoking history. That combination has also demonstrated improved 2 year OS in that subset of patients compared to historical controls (72% *vs* 60% 2 year OS) (272). Other classes of DDR inhibitors such as WEE1 (Adavosertib), ATM, and DNA-PK inhibitors are currently being tested in phase I trials either in conjunction with radiation alone or chemoradiation in multiple disease sites. A recently completed phase I study evaluated Adavosertib in combination with RT and full-dose gemcitabine for 34 patients with locally advanced pancreatic cancer (273). In that study, median OS was 21.7 months which compares favorably with that of patients treated in the LAP07 trial (11.9–13.6 months), which had similar eligibility criteria and used gemcitabine (274). This sets Adavosertib as a promising drug in terms of clinical development compared to PARP inhibitors. The DNA-PKc inhibitor M3814 (Peposertib) has demonstrated promising anti-tumor activity in a recently published phase Ia study and is currently being tested concurrent with radiation in at least four phase I clinical trials covering different disease sites and different radiation fractionation regimens (275). ATM, ATR, and CHK1 inhibitors are also currently in several early phase clinical trials. Taken together, validating biomarkers of response for these novel agents to identify the subset of patients who will derive the most benefit and the most acceptable toxicity in return remains to be a challenge (276).

Nanotechnology offers a new area of exciting research where nanoparticles can be used for targeted radiotherapy, either as sensitizers of external beams or as delivery vehicles for therapeutic radionuclides (277). In a phase II/III study, NBTXR3, a first-in-class radiosensitizer hafnium oxide nanoparticle, which is activated by radiation therapy, a significantly higher pathologic complete response was observed in the patients whose soft tissue sarcomas were injected with NBTXR3 prior to radiation compared to those who were not. There was no significant difference in toxicity between the two groups and no treatment-related death occurred (278). Although this is very promising data in the sarcoma field where very few patients achieve pathologic complete response with preoperative radiation and possibly in other cancers as well, a lot of challenges lie ahead for the clinical implementation of this technology and overcoming its limitations, particularly optimization of delivery (279).

The PACIFIC trial has revolutionized the management and therefore the outcomes of patients with locally advanced NSCLC. It has also set unprecedented clinical evidence supporting the interplay of chemoradiation and immunotherapy (280, 281). Importantly however it has posed many pressing questions regarding the optimal dosing, sequencing, and safety of combining radiation with immunotherapy. Currently, a plethora of clinical trials are attempting to answer those questions. Recently, the DETERRED trial demonstrated the safety and efficacy of adding Atezolizumab (anti-PD-L1) concurrently with chemoradiation (282) as well as the Phase 2 KEYNOTE-799 with concurrent delivery of Pembrolizumab (anti-PD-1) and radiation in locally advanced NSCLC (283). It will therefore be important to compare that regimen with the PACIFIC regimen where Durvalumab (anti-PD-L1) was given after chemoradiation in the consolidation setting. In head and neck cancer, a number of phase I/II clinical trials are testing the feasibility of combining chemoradiation with immunotherapy in the definitive setting. Collectively, those early studies have demonstrated the safety of the combination (284–287). A recent report by Weiss et al. showed that concurrent definitive immunoradiotherapy for patients with stage III-IV head and neck cancer who are ineligible for cisplatin had 24-month PFS and overall survival rates were 71% which exceeded their primary hypothesis (288). However, a substantial clinical benefit is yet to be proven in the phase III setting.

In the metastatic setting, several prospective trials have been conducted to test the abscopal effect of radiation, which stems from many anecdotal reports and arguably stimulated much of the hype regarding the combination of radiation and immunotherapy (289). The abscopal effect of radiation refers to the shrinkage or disappearance of sites of metastasis that were not directly treated with radiation. Although the mechanisms of this observation are still being elucidated, it is believed that the addition of immunotherapy to radiation regimens allows the immune system to mount a more systemic response against the tumor. PEMBRO-RT is a phase II study which asked the question whether stereotactic body radiotherapy (SBRT) enhances the effect of immune checkpoint inhibition in nonirradiated lung cancer lesions in metastatic NSCLC. In that study, patients with metastatic NSCLC were randomized to receiving pembrolizumab either alone or after SBRT, which was delivered to a single tumor site. There was a trend towards better overall response (ORR) and improved PFS in the combination arm but did not reach statistical significance. Interestingly, the benefit was more evident in patients with PD-L1 negative tumors and in subgroup analysis, improved ORR and PFS reached statistical significance in that group of patients (290). This again highlights the importance of discovering and understanding what molecular markers are important in the response to RT alone and in combination with targeted agents. In metastatic head and neck cancer, a similar phase II study randomized patients to either Nivolumab (anti-PD-1) alone or after SBRT to one metastatic site. Unfortunately the study did not find improvement in response, PFS, or OS between the two arms and there was no evidence of an abscopal effect with the addition of SBRT to Nivolumab in unselected patients with metastatic HNSCC (291). Interestingly however, in the neoadjuvant setting in early stage resectable NSCLC, concurrent SBRT and Durvalumab was safe and associated with significantly better pathological response compared to neoadjuvant Durvalumab alone demonstrating a robust evidence of abscopal immune-modulatory effect of radiation (292). These contrasting results could probably be attributed to the hypothesis that immunotherapy is generally more effective with less disease burden and therefore the abscopal effect could be captured in that setting (293). Taken together, phase III data is needed to validate the combinatorial benefit of radiation and immunotherapy in the metastatic setting and also better defining correlates of response based on biomarkers.

As outlined above there are many clinical trials testing different radiosensitization paradigms. That is not meant to be a comprehensive list but rather to paint a picture for the diverse nature of signaling mechanisms that could potentially be targeted to improve the therapeutic ratio of radiation. Importantly, while there are examples of successful radiation-targeted therapy combination in clinic, failures certainly outweigh those few successes. Therefore, a lot remains to be done in to decrease attrition rates of novel radiosensitizers in the clinic.

## The Challenges Ahead for Clinical Implementation

**Figure 3:**
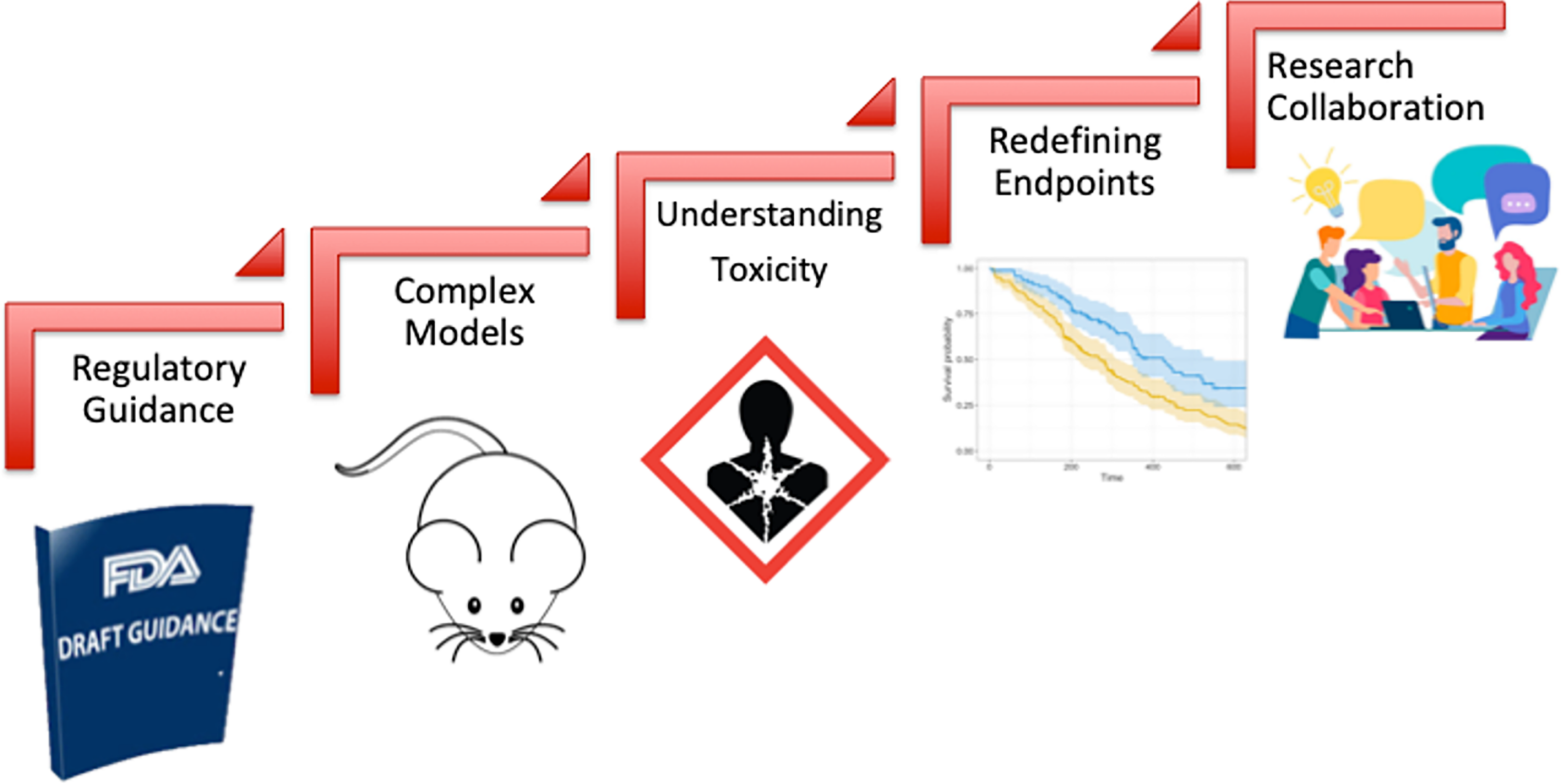
Challenges of clinical development of novel drug-radiotherapy combinations.

Oncology drug development has witnessed a significant growth over the last decade that was coupled with improved cancer outcomes and unprecedented drop in cancer related death rates (294). However, the development of novel radiosensitizers lagged behind reflecting lack of incentive by pharmaceutical industry to invest in this pipeline. This huge gap led to holding a collaborative workshop by the FDA-AACR-ASTRO in 2018 to bring together various stakeholders including representatives of academia, industry, patient advocacy groups and the FDA to identify key challenges and design a roadmap for bridging this gap (104). This effort was also preceded by similar efforts in the UK highlighting the importance of this issue in the overall goal of improving cancer control rates where radiation therapy plays a central role as a curative and palliative treatment (105). As highlighted in **Figure 3**, the main challenges identified were: (1) lack of regulatory guidance by the FDA detailing the approval pathway for drug-radiotherapy combination particularly with regard to the extent of required preclinical data, (2) choice of adequate model systems that can reflect tumor complexity and heterogeneity and enable testing various radiation techniques and schedules, (3) complexity of the definition of ‘safety’ in the radiation setting as it should take into account normal tissue toxicity and long term toxicity which are not traditionally considered in drug only studies, (4) perceived impracticality of traditional clinical trial regulatory endpoints (such as OS and PFS) when testing novel drug-radiotherapy combinations particularly in the curative setting and finally (5) historically limited collaboration among medical and radiation oncologists particularly in the United states which is crucial for aligning research perspectives and goals. Moving forward, overcoming these hurdles and prioritizing communication among key stakeholders in the field will be crucial to propel the radiosensitizer pipeline. The year 2020 was arguably a landmark year for drug-radiotherapy combinations, with two novel radiosensitizers getting fast track and breakthrough designations: NBTXR3 and Debio 1143 respectively (294). However, the field is yet to witness new market approvals as we strive to overcome challenges and improve patient outcomes.

## Author Contributions

ME and NC contributed equally to the writing and planning of the content of the review. RR provided clinical input and expertise to the review and MF edited and directed the writing and content within the review. All authors contributed to the article and approved the submitted version.

## Funding

MF was supported by grants from the National Institute of Health and National Cancer Institute R01CA167291, R01CA211098, R01 CA254110. NC and MF were also supported by NIH/NCI grant U01HL143403. MF was additionally supported by the Riley Children’s Foundation.

## Conflict of Interest

The authors declare that the research was conducted in the absence of any commercial or financial relationships that could be construed as a potential conflict of interest.

## Publisher’s Note

All claims expressed in this article are solely those of the authors and do not necessarily represent those of their affiliated organizations, or those of the publisher, the editors and the reviewers. Any product that may be evaluated in this article, or claim that may be made by its manufacturer, is not guaranteed or endorsed by the publisher.
